# The geno-spatio analysis of *Mycobacterium tuberculosis* complex in hot and cold spots of Guangxi, China

**DOI:** 10.1186/s12879-020-05189-y

**Published:** 2020-07-01

**Authors:** Dingwen Lin, Zhezhe Cui, Virasakdi Chongsuvivatwong, Prasit Palittapongarnpim, Angkana Chaiprasert, Wuthiwat Ruangchai, Jing Ou, Liwen Huang

**Affiliations:** 1 Department of Tuberculosis Control, Guangxi Zhuang Autonomous Region Center for Disease Control and Prevention, Nanning, 530028 Guangxi China; 2 Epidemiology Unit, Faculty of Medicine, Prince of Songkla University, Songkhla, 90110 Thailand; 3 Pornchai Matangkasombut Center of Microbial Genomics, Department of Microbiology, Faculty of Science, Mahidol University, Bangkok, 10700 Thailand; 4 Office for Research and Development, Faculty of Medicine Siriraj Hospital, Mahidol University, Bangkok, 10700 Thailand

**Keywords:** Tuberculosis, Genotypes, Polymorphisms, Spatial, Influence

## Abstract

**Background:**

At present, there are few studies on polymorphism of *Mycobacterium tuberculosis* (Mtb) gene and how it affects the TB epidemic. This study aimed to document the differences of polymorphisms between tuberculosis hot and cold spot areas of Guangxi Zhuang Autonomous Region, China.

**Methods:**

The cold and hot spot areas, each with 3 counties, had been pre-identified by TB incidence for 5 years from the surveillance database. Whole genome sequencing analysis was performed on all sputum Mtb isolates from the detected cases during January and June 2018. Single nucleotide polymorphism (SNP) of each isolate compared to the H37Rv strain were called and used for lineage and sub-lineage identification. Pairwise SNP differences between every pair of isolates were computed. Analyses of Molecular Variance (AMOVA) across counties of the same hot or cold spot area and between the two areas were performed.

**Results:**

As a whole, 59.8% (57.7% sub-lineage 2.2 and 2.1% sub-lineage 2.1) and 39.8% (17.8% sub-lineage 4.4, 6.5% sub-lineage 4.2 and 15.5% sub-lineage 4.5) of the Mtb strains were Lineage 2 and Lineage 4 respectively. The percentages of sub-lineage 2.2 (Beijing family strains) are significantly higher in hot spots. Through the MDS dimension reduction, the genomic population structure in the three hot spot counties is significantly different from those three cold spot counties (T-test *p* = 0.05). The median of SNPs distances among Mtb isolates in cold spots was greater than that in hot spots (897 vs 746, Rank-sum test *p* < 0.001). Three genomic clusters, each with genomic distance ≤12 SNPs, were identified with 2, 3 and 4 consanguineous strains. Two clusters were from hot spots and one was from cold spots.

**Conclusion:**

Narrower genotype diversity in the hot area may indicate higher transmissibility of the Mtb strains in the area compared to those in the cold spot area.

## Background

It is widely recognized that *Mycobacterium tuberculosis* (Mtb) of different molecular types have different transmission capacities, pathogenicity and drug resistance rates [1, 2]. Therefore, Mtb genotypes may be associated with the tuberculosis (TB) endemicity [3, 4]. Guangxi Zhuang Autonomous Region is a southern region of China with a seriously high TB prevalence [5]. However, the internal TB situation in Guangxi varies greatly. Previous spatiotemporal studies found a significantly high notification spatial cluster (hot spots) and a significantly low notification spatial cluster (cold spots) through spatiotemporal scanning technology [6]. Some environmental and socioeconomic status factors related to the TB epidemics in this region have been identified. However, the molecular biological explanations for this situation are still lacking.

Whole genome sequencing (WGS) was employed in this study because it provided a powerful tool for phylogenetic analysis and epidemiological tracing than other conventional methods such as IS*6110* restriction fragment length polymorphism (RFLP), spoligotyping, and variable-number tandem repeat (VNTR) typing [7–11]. Single nucleotide polymorphism (SNP) based phylogenetic networks of Mtb strains have been used to identify the super-spreaders and transmission [12]. However, the relationship between the number of SNP difference and geographic spread of Mtb has never been studies in depth. Such a study would be feasible in our study since information on Mtb genome of the population and geographic information of the TB case is available.

Based on the above reasons, this study was conducted to 1) document the differences of Mtb polymorphisms (SNP) between hot and cold spot areas, 2) identify Mtb genotype with high level of local transmission and 3) analyse the relationship between different level of SNP variations and geographic distribution of the Mtb isolates.

## Methods

### Study design

A case-only study in the TB notification hot and cold spot areas of Guangxi was performed from January to June 2018.

### Study setting

Based on the spatial clustering analysis, three counties (C1-C3) with significantly high TB notification were identified as hot spots, and three counties (C4-C6) with significantly low notification rate were identified as cold spots [6]. The permanent population (Unit: Thousand) of these counties are 536.2 (C1), 325.6 (C2), 604.2 (C3), 720.7 (C4), 1191.8 (C5) and 795.4 (C6). In these six study sites, patients who were suspected to have pulmonary TB were confirmed at the designated hospitals for TB with chest radiography, sputum smear and culture. All Mtb isolates from the culture were shipped to the Guangxi center for disease prevention and control (GXCDC) where Mtb deoxyribonucleic acid (DNA) extraction was performed before transporting the DNA to Zeta Biosciences company (Shanghai) for WGS and upstream data analysis. After receiving informed consents, epidemiological investigations of the TB patients were conducted by local hospital teams under the supervision of GXCDC. Clinical and other laboratory data of the recruited patients were retrieved from routinely entered data of the National Notifiable Disease Reported System which was overseen by GXCDC.

### Study participants and selection methods

#### Sample size

The sample size of this study was calculated based on the formula of two independent proportions comparison [13]. According to a previous study of dominant genotype (Beijing family strains) in Guangxi, the estimated percentage of Beijing strains was 70% in hot spot areas and 50% in cold spot areas. With a type I error of 0.05 and a power of 90%, at least 248 active TB cases (124 cases in each spots) were required.

#### Eligibility criteria for index cases

Eligible index TB cases must have been a resident in the study sites for at least 2 years prior to TB diagnosis. Isolates from individuals who were unable to communicate with investigators and children under the age of five were excluded.

### WGS performance and SNP calling

At the GXCDC, Mtb DNA extraction was conducted by a genetic sample kit (HiPure Bacterial DNA Kit, Magen Biotech Co. Ltd). At Zeta Biosciences (Shanghai), a WGS kit was employed to obtain enough nucleic acid for the sequencing of the downstream analysis. Each sample was quantified by Qubit 2.0 Fluorometer (Invitrogen, Carlsbad, CA, USA). Next generation sequencing library with 350-base-pair (bp) paired-end preparations were constructed for each purified DNA sample according to the manufacturer’s criterion (Illumina TruSeq DNA Nano Library Prep Kit). Then libraries with different indices were multiplexed and loaded on an Illumina HiSeq instrument with an expected coverage of 100 following the manufacturer’s instructions (Illumina, San Diego, CA, USA). After analyzing a short sequence alignment of the Mtb according to the reference genome H37Rv, multiple sequence alignment test strains of SNPs/InDels were used to obtain the corresponding molecular classification [14]. A Burrows-Wheeler transform algorithm and genome analysis toolkit packages (GATK v 4.1.1.0, Broad Institute, USA) were applied in this process. The SNVs that were present in any drug-resistance gene, mobile genetic element, phage, PE/PPE region and non-homozygous SNVs were discarded. The remaining SNVs had been converted to an SNV-supermatrix using an in-house Python script before being used in the phylogenetic analysis. Phylogenetic trees were constructed by Bayesian Inference (BI) methods using MrBayes [15]. The BI tree was supported with posterior probabilities. The tree was visualized by FigTree version 1.4.2. Molecular typing and statistical inference were conducted based on the genotype assignment of the isolates were based on SNPs classification as previously published [16] Finally, the crude and filtered fasta-files, vcf files, SNPs distance matrix, classified spoligotype and lineage genotype data, and the Weir and Cockerham weighted genetic group structure differentiation coefficient (F-statistics, Fst) between the subgroups were delivered to the GXCDC for downstream data analysis.

### Data management and analysis

Epi Data (v 3.1) was used for double entering the data at regular intervals while R (v 3.3.2) was used for epidemiological data management and analysis. MEGA-X (v 10.0.5) and Fig Tree (v 1.4.4) were used to build phylogenetic trees with color labels. Multidimensional scaling model (MDS) was employed to test the between and within group of differentiation *Fst* value from Analysis of Molecular Variance (AMOVA) by reducing the matrix dimensions. Clustering of isolates were analyzed based on several levels of SNP distances starting from 12 as generally done but were also done at the SNP distances of 24, 48 and 96, and so on. Isolates with less than 12 SNP distances were considered as recent epidemiologically linked. Clusters of isolates with higher distances were analyzed in regard to geographical proximity. Cluster of isolates having SNP difference within each of these cut points were identified and checked to see whether all clusters members were from the same county, or area (Hot and cold spots). Rank-sum test, a non-parametric analysis, was employed to compare the median of pairwise SNPs distance between two groups.

## Results

**Fig. 1:**
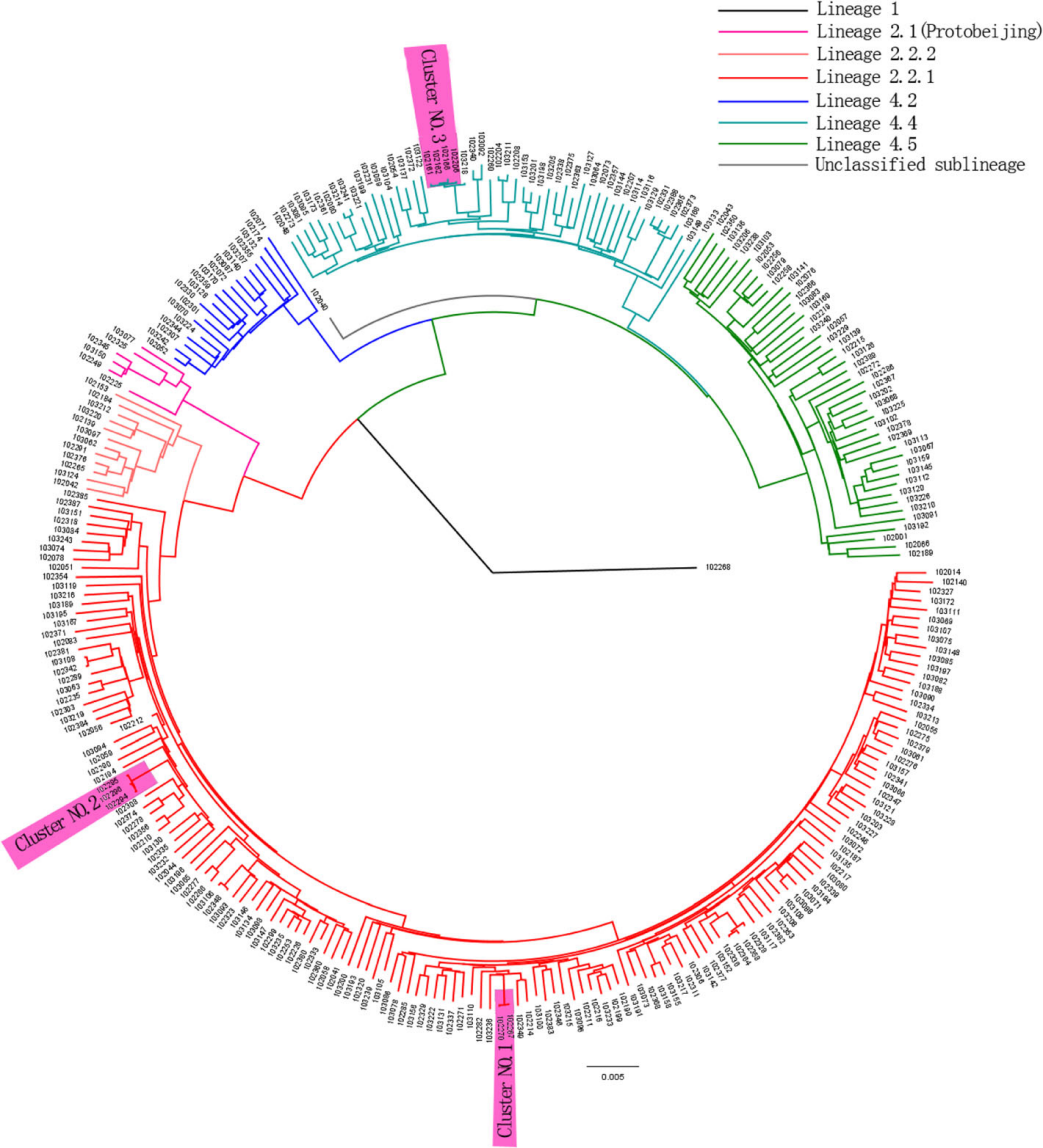
The phylogenetic tree of Mtb constructed by the Bayesian Inference method. The three most likely recent clusters,with the criterion of SNP distances less than or equal to 12, were shaded. The clusters No. 1 & 2 were found in a county of hot spots and the cluster No. 3 was found in two counties of cold spots. Only a single Lineage 1 isolate (102268) was identified

After excluding 13 participants (8 patients with non-tuberculosis mycobacteria infection and 5 patients with low quality of Mtb DNA), a total of 147 isolates from hot spots and 144 from cold spots were included for further analysis. Their phylogenetic trees are shown in Fig. 1. The predominate lineage is lineage 2 (59.8%), with its major sub-lineage 2.2 or the Beijing family (57.7%). The percentage of sub-lineage 2.1 (2.1%) was relatively high compared to other part of China as previously reported [17]. Other major genotype included lineage 4 (39.8%) with its sub-lineage 4.4 (17.8%), 4.2 (6.5%) and 4.5 (15.5%). Three most likely clusters were detected at the criteria of SNPs distance less than or equal to 12.

Table 1 shows the lineage distribution in each county while Table 2 compared the Beijing family with all other strains. The proportion of lineage 2.2.1 and 2.2.2 (Beijing family strains) was evenly distributed in the three hot counties, and was significantly higher than that in the cold counties (*p* = 0.022). Compared with the hot spots, the isolates from the cold spots are more genetically diverse. There was no significant difference of Beijing family percentages between the counties in each spot (*p* = 0.361 and 0.482).

**Table 1:** Lineage distribution at county level(n, row%)

Region	Subgroups	lineage1.1.1.1	lineage2.1	lineage2.2.1		lineage2.2.2	lineage4.2.2	lineage4.4.1	lineage4.4.2	lineage4.5	Total
				Ancestral	Modern						
Hot spots	C1	0 (0)	1 (1.5)	14 (21.54)	27 (41.54)	2 (3.1)	2 (3.1)	0 (0)	8 (12.3)	11 (16.9)	65 (100)
	C2	0 (0)	1 (2.4)	9 (21.95)	13 (31.71)	1 (2.4)	4 (9.8)	0 (0)	10 (24.4)	3 (7.3)	41 (100)
	C3	1 (2.4)	3 (7.3)	17 (41.46)	10 (24.39)	2 (4.9)	2 (4.9)	0 (0)	1 (2.4)	5 (12.2)	41 (100)
Cold spots	C4	0 (0)	0 (0)	12 (20.34)	18 (30.51)	3 (5.1)	3 (5.1)	0 (0)	12 (20.3)	11 (18.6)	59 (100)
	C5	0 (0)	1 (1.7)	9 (15.52)	15 (25.86)	2 (3.4)	5 (8.6)	1 (1.7)	14 (24.1)	11 (19)	58 (100)
	C6	0 (0)	0 (0)	9 (33.33)	3 (11.11)	2 (7.4)	3 (11.1)	0 (0)	6 (22.2)	4 (14.8)	27 (100)
Total		1 (0.3)	6 (2.1)	70 (24.05)	86 (29.55)	12 (4.1)	19 (6.5)	1 (0.3)	51 (17.5)	45 (15.5)	291 (100)

**Table 2:** Comparison the proportion of Beijing genotype in each group

Region	Subgroups	Beijing	Non-Beijing	*p* value (Compare counties in each spots)	*p* value (Compare hot and cold spots)
Hot spots	C1	43	22	0.361	0.022
	C2	23	18		
	C3	29	12		
Cold spots	C4	33	26	0.482	
	C5	26	32		
	C6	14	13		

**Fig. 2:**
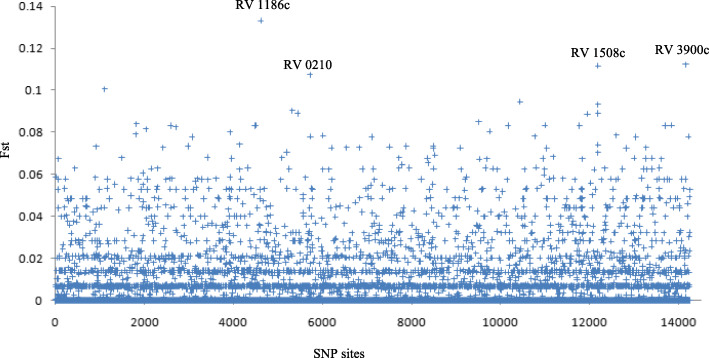
The Fst of each SNP sites of MTB isolates in hot spots compared with cold spots (Weir and Cockerham weighted). The SNP sites that showed highest Fst are labeled

As shown in Fig. 2, after AMOVA computing and filtering, 14,250 SNP sites were kept for Fst estimation between hot and cold spots. The average Fst value with Weir and Cockerham weighted is 0.0195. The SNP sites which have the highest Fst estimation are within the coding sequences of Rv1186c (PruC) (0.133), and hypothetical proteins, Rv0210 (0.101), Rv1508c (0.1074) and Rv3900c (0.112). PruC is a membrane-associated DNA-binding protein that control proline metabolism.

**Fig. 3:**
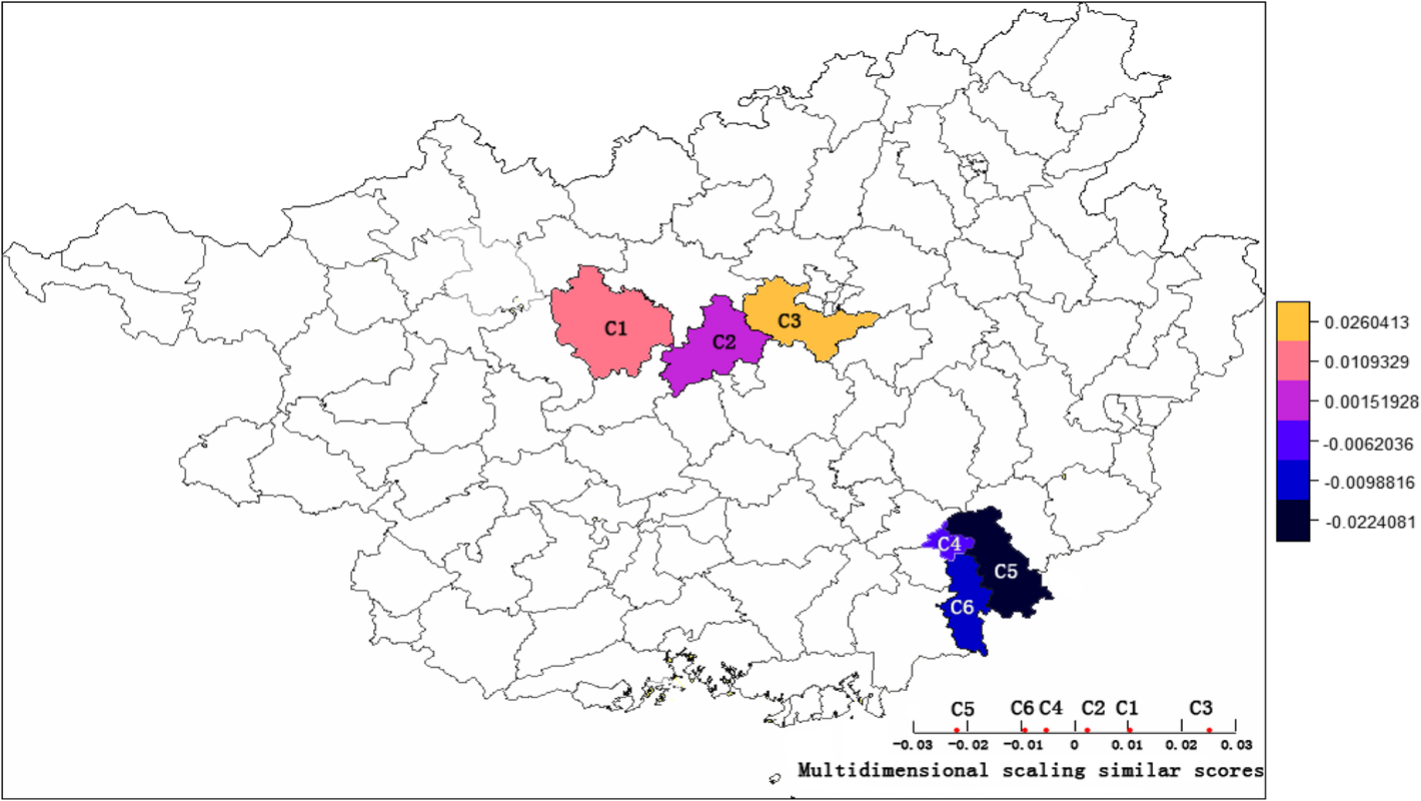
The Multidemensional scaling similar scores of Fst between six counties. (We are very grateful to Center for Spatial Sciences at the University of California, Davis for providing us with the map files which are freely available for academic use. https://www.gadm.org/about.html)

The Multidimensional Scaling analysis was used to compare the Fst of tuberculosis strains across six counties as shown in Fig. 3. After introducing the Fst matrix, we performed the MDS and reduced the distance dimension to one to see the similar scores between these counties, and mapped it. The Fst distance similarity scores have certain clustering property according to different spatial spots. The mean similarity scores of three hot spot counties is 0.013 ± 0.012, while the mean score for the three cold spots was − 0.013 ± 0.008. The difference between two groups is significant (*p* = 0.05).

**Fig. 4:**
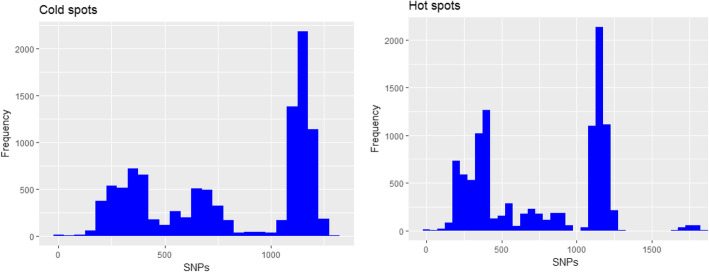
The frequency distribution of SNPs distances in hot and cold spot areas. In general, SNP distance peak of > 1000 are form differences between isolates belonging to different lineages, in this case between L2 and L4. 500–1000 are resulted from major sub-lineages such as between L2.1 and L2.2. The ones < 500 are usually from differences between more detailed sub-lineages such Asian African2 and Asian African 3. < 100 is almost definitely from the difference between isolates belonging to the same detailed sub-lineages or the same cluster

Among 42,195 pairs of SNPs difference, the shortest distance between these stains is 7 SNPs, and the longest distance pair was 1893 SNPs. The average distance was 703 SNPs. Figure 4 shows the frequency of SNPs distance (one to one comparison) in hot and cold spot areas. Both cold and hot spots had three peaks of SNPs distances in the range of 0–500, 501–1000 and > 1000. The leftmost peaks included the distances of recently transmitted isolates. The median of SNPs distance among isolates from cold spots is greater than that in hot spot areas (897 vs 746, Rank-sum test *p* < 0.001). This is consistent with the higher transmission activities in the hot spot.

Table 3 shows the relationship of SNPs based clusters within and across the hot and cold spots at different SNPs level. Two isolates having SNPs difference of less than or equal to 12 were likely to be related by recent transmission. We detected two genetic clusters with 2 and 3 consanguineous strains in a hot spot county and one genetic cluster of 4 consanguineous strains cross two cold spot counties (Supplementary Table 1.). Only if the clustering criterion was relaxed to 96 of SNPs distance, we would identify the 3 more consanguineous strains clusters across two zones. The number of clusters, geometric mean of cluster size, number of cluster crossing the county and number of cluster crossing the zone have positive correlations with cut point level (*p* < 0.05).

**Table 3:** SNPs based clustering at different clustering criterias

SNP difference cut point	≤ 12	≤ 24	≤ 48	≤ 96	≤ 192	≤ 384
No. of clusters	3	6	8	20	16	27
Geometric mean (geometric SD) of cluster size	2.88 (0.82)	1.92 (1.17)	2.48 (1.06)	2.38 (1.23)	5.87 (15.90)	36.77 (57.12)
No. of cluster crossing counties in the same zone	1	4	4	10	13	24
No. of cluster acrossing zones	0	0	0	3	13	24

## Discussion

This study identified the molecular biological characteristics of Mtb in different TB epidemic areas of southern China. Only two major lineages (L2 and L4) were found in this study setting. The predominate Mtb strain is lineage 2.2 (Beijing family), and it was significantly higher in TB notification hot spots. Through the population gene structure analysis (AMOVA) and SNPs comparison between cold and hot spots and the multidimensional scaling modeling of each county, we found that the two spot areas had some differences in genetic structure, and the spatial internal consistency was relatively high. Specific SNPs sites between the cold and hot spots with high Fst estimation mapped to special proteins that may contribute to the pathogenicity differences in Mtb. Three genomic (SNPs ≤12) and geographic groups were detected and identified as Mtb recent transmission individuals.

Previous studies have suggested that *Mtb* among human originated in Africa and was divided into seven lineages by several thousand years of mutations [18–21]. The evolution of Mtb has been related to human migration and evolution. It spread from Africa to the rest of the world along with human migration and formed the current genotype distribution. Nowadays, the most prevalent Mtb strain in China is lineage 2 [22]. Although in northern China, the proportion of Beijing strains is as high as 80%, which were mostly Modern Beijing strains), as the latitude decreases, this proportion decreases [23]. Moreover, with the increase of population mobility, the polymorphism of *Mtb* genotype becomes more and more obvious. Therefore, as a southern province of China, Guangxi has more proportion of Ancestral Beijing strains and genetic diversity of Mtb strains than that in northern region [24]. The origin of Protobeijing strain (L 2.1) is likely to be in Southern China as it has the highest percentage [16]. In this study, the lineage 4 with three sub-lineages (L4.2, L4.4 and L4.5) also accounted for a large proportion. In contrast to other major human-adapted lineages, lineage 4 appears with significant frequency on all inhabited continents [25]. Thus, it is the most widespread cause of TB in humans geographically [26]. Among this lineage, L4.4 and L4.5 were mostly reported from China, although we usually called L4 as Euro-American [27]. Stucki hypothesized that the global spread of L4 maybe caused by European migration and colonization [27]. Yet, the reasons for this spatial distribution in China needs more evidence.

In this study, by comparing all the gene loci of the cold and hot spots strain, it was found that the two populations had mutation differences in some special regulatory proteins. The mutation of Rv1186c (PruC) has a certain significance. Mtb is an obligate aerobic bacterium that needs oxygen to grow. However, paradoxically, it shows a remarkable metabolic flexibility that allows itself to survive and metabolize in oxygen-deprived conditions [28]. It has been shown that mycobacteria can grow on proline as the sole carbon and energy source under hypoxia, and it is regulated by a unique transcriptional regulator (PruC) [29]. An animal study performed by Smith DA et al. found that mycobacteria with abnormal proline metabolism were nonpathogenic in immune-competent mice [30]. However, Rv1186c was predicted to be non-essential gene in the papers of DeJesus et al. [31] and Lamichhane et al. [32], except the paper of Sassetti et al. [33]. Apart from Rv1186c, the 3 genes (Rv0210, Rv1508c, Rv3900c) that came out of the SNP analysis are unknown function genes and all are predicted as non-essential genes. Thus, it is expected that there will be no significant association with the pathogenicity of Mtb. Nevertheless, further epidemiological and clinical exploration are needed.

As the dominant genotype, Beijing family strains have been shown to cluster more frequently [34]. This suggests that Mtb recent transmission is more likely to occur in such strains [35, 36]. Some scholars claimed that the determination of recent transmission or MTB outbreak (transmission within 2 or 3 years) is that the cut-offs of WGS-Based genomic distance is less than or equal to 12 SNPs [37]. However, only three recent transmission groups in this study were detected. In the previous research, the research samples with recent transmission cases are generally from communities of long-term surveillance or tuberculosis outbreaks field [12, 38]. Although the specimens in this study were from two spatial clusters of TB notification (hot and cold spots), it is likely to be true that there is no obvious outbreak occurring during the study period. The locations of included participants were scattered. Thus, this study showed that the median SNPs distance of strains in hot spots was significantly lower than that in cold spots. The comparison of SNPs population genetic structure was also proved the significant difference in the gene structure between the two areas, but the differences within the areas were relatively small. We did not find any cluster that have members crossing the hot and cold spots. Actually the minimal SNP distance between any genetically related isolates in both spots were at least 96. This suggests that the transmission pattern of the Mtb in hot spots may be different from those in cold spots. Local transmission in hot spot areas (over a period of more than 3 years) is more likely than in cold spot areas. Homologous transmission may occur over a longer period of time [37]. They might have gotten the same type of *Mycobacterium tuberculosis* many years ago, and the strains might have mutated after a long time of latent infection, proliferation and then endogenous reactivation. Thus, the SNPs distance between the two strains would become larger. Our study also performed contact tracing among included cases [39]. The results showed that the detection rate of TB in household contacts was very low. Only two domestic cases have been detected in hot spot areas. Therefore, we believe that the transmission patterns of TB patients in cold and hot spots were dominated by community transmission.

Either the spreading of the Mtb was local or there were some socioeconomic factors that hinder the transmission between the hot and cold spot areas. Our previous studies on the ecology of tuberculosis suggested that there is a negative correlation between average sunshine time and reported incidence of tuberculosis [6]. Therefore, there may be some interaction between natural factors and strain pathogenicity. This requires further exploration.

Meanwhile, sub-lineage 2.1 (Protobeijing strain), a special subgroup related to drug resistance, is mainly concentrated in hot spots. Since it has been reported that the virulence and drug resistance of Beijing gene strain is greater than other strains [34, 40, 41], we can infer that the prevalence of this strain in hot spots is one of the important reasons for its higher TB epidemic than cold spots.

The limitation of this study is that the sputum isolates were collected mainly from passively detected cases in the public hospital. This may have contributed to the fact that some specimens of the TB cases treated in private practice were not included during the study period, which caused the bias to some extent. However, as TB case management strategies become more widely publicized under the national TB control programme, this impact should diminish [42, 43]. The estimated inclusion rate were over 90%.

## Conclusions

Mtb genotype distribution is associated with TB incidence. Hot spot area in Guangxi is associated with Beijing family predomination. Narrower genotype diversity in the hot area may indicate higher transmissibility of the Mtb strains in the area compared to those in the cold spot area. These findings demonstrate that promotion of the genetic diagnosis in tuberculosis clinics and early identification of Beijing family strain should be considered in tracking and stopping TB transmission.

## Supplementary information

**Additional file 1: Supplementary Table 1.** Genetic clustering based on the SNPs (≤12)

## Data Availability

The raw sequence data reported in this paper have been deposited in the Genome Sequence Archive (Genomics, Proteomics & Bioinformatics 2017) in BIG Data Center (Nucleic Acids Res 2019), Beijing Institute of Genomics (BIG), Chinese Academy of Sciences, under accession numbers PRJCA002021 that are publicly accessible at https://bigd.big.ac.cn/gsa.

## Abbreviations

AMOVA: Analyses of Molecular Variance
BI: Bayesian Inference
CDC: Center for DiseaseControl and Prevention
DNA: deoxyribonucleic acid
Fst: F-statistics
MDS: Multidimensional scaling model
Mtb: *Mycobacterium tuberculosis*
RFLP: Restriction Fragment Length Polymorphism
SNP: Single Nucleotide Polymorphism
TB: Tuberculosis
VNTR: Variable-Number Tandem Repeat
WGS: Whole genome sequencing.
