# Supplementary material for: The geno-spatio analysis of Mycobacterium tuberculosis complex in hot and cold spots of Guangxi, China
Source: BMC Infect Dis. 2020 Jul 1;20:462. doi: 10.1186/s12879-020-05189-y (PMC7329418; doi:10.1186/s12879-020-05189-y)
Supplement: Supplementary file 1 — Additional file 1: Supplementary Table 1. Genetic clustering based on the SNPs (≤12) [file 12879_2020_5189_MOESM1_ESM.docx]

| **Supplementary Table 1. Genetic clustering based on the SNPs (≤12)** | | | | | | |
| --- | --- | --- | --- | --- | --- | --- |
| **County** | **Lineage** | **SNPs Matrix** | | | | |
| C3 | L 2.2.1 | Cluster 1 | 102267 | 102270 |  |  |
|  |  | 102267 | - | 7 |  |  |
|  |  | 102270 | 7 | - |  |  |
|  |  |  |  |  |  |  |
| C3 | L 2.2.1 | Cluster 2 | 102294 | 102295 | 102296 |  |
|  |  | 102294 | - | 11 | 12 |  |
|  |  | 102295 | 11 | - | 15 |  |
|  |  | 102296 | 12 | 15 | - |  |
|  |  |  |  |  |  |  |
| C5 | L 4.4.2 | Cluster 3 | 102162 | 102206 | 102166 | 102161 |
|  |  | 102162 | - | 9 | 11 | 11 |
|  |  | 102206 | 9 | - | 16 | 12 |
|  |  | 102166 | 11 | 16 | - | 16 |
| C6 |  | 102161 | 11 | 12 | 16 | - |
